# Immediate Unrestricted Assisted Full Weight-Bearing After Bicondylar Tibial Plateau Fixation: A Case Series

**DOI:** 10.7759/cureus.115852

**Published:** 2026-09-06

**Authors:** Pratyaksh K Gupta, Ritabh Kumar, Vineet Dabas

**Affiliations:** 1 Orthopaedic Surgery, Indian Spinal Injuries Centre, New Delhi, IND; 2 Orthopaedic Surgery, Bharati Vidyapeeth (Deemed to be University) Medical College, Pune, IND; 3 Orthopaedics, Indian Spinal Injuries Centre, New Delhi, IND; 4 Orthopaedics and Trauma, Indian Spinal Injuries Centre, New Delhi, IND

**Keywords:** bicondylar fracture, dual-plate fixation, immediate full weight-bearing, schatzker types v-vi, tibial plateau fracture

## Abstract

High-energy bicondylar tibial plateau fractures of Schatzker types V and VI are conventionally managed, after open reduction and internal fixation, with 6-12 weeks of restricted weight-bearing, out of concern for loss of reduction and articular collapse. Emerging evidence questions whether this restriction is necessary. Reports of immediate weight-bearing specifically after fixation of these most unstable bicondylar patterns remain scarce, as most comparative studies pool this subtype within mixed cohorts. We present three consecutive patients (two men and one woman; mean age: 54 years; all of North Indian ethnicity) with closed, high-velocity Schatzker type V-VI tibial plateau fractures sustained in road traffic accidents. Each was treated with open reduction and internal fixation using a dual-plate or three-plate construct, with allograft impaction of the metaphyseal void created after the elevation of the depressed articular fragments. In departure from the conventional restricted protocol, each patient was mobilised to full weight-bearing as tolerated from the first postoperative day, using a walking aid for balance rather than for offloading, with early active range-of-motion exercises and no brace. At six weeks, all three patients had achieved independent ambulation with a walking aid, knee flexion beyond 90 degrees, no pain during activities of daily living, and a clinically acceptable gait. Follow-up radiographs showed no loss of fixation, screw cut-out, or articular collapse, and no patient sustained a wound complication, infection, or revision surgery. Immediate unrestricted full weight-bearing after stable plate fixation of Schatzker type V-VI tibial plateau fractures appears safe and may accelerate functional recovery. These observations are concordant with recent randomised and comparative literature and are underpinned by a coherent bone-biological rationale in which physiological loading promotes rather than jeopardises union. This report supports a considered shift away from routine prolonged non-weight-bearing towards individualised, stability-guided early mobilisation, even for the most unstable bicondylar patterns, provided the fixation construct is intraoperatively judged sound. Larger prospective comparative studies are needed to confirm these findings and refine patient selection.

## Introduction

High-energy tibial plateau fracture patterns, most notably Schatzker type VI fractures defined by metaphyseal-diaphyseal dissociation with variable condylar comminution, typically result from road traffic accidents [1]. Open reduction and internal fixation (ORIF) with locking plates, often two or more plates for bicondylar patterns, has become the standard treatment. Postoperative weight-bearing protocols have, however, remained far less standardised than the surgical technique itself.

Surgeons have historically recommended 6-12 weeks of non-weight-bearing after fixation, out of concern that axial load will cause loss of reduction before bony consolidation. This is reflected in AO (Arbeitsgemeinschaft für Osteosynthesefragen) guidance recommending toe-touch weight-bearing for 6-8, and up to 12, weeks. Yet surveys show striking inconsistency in practice: in one national survey, only 11.7% of surgeons recommended immediate weight-bearing, while more than 55% restricted it until six weeks [2]. Over the past decade, a growing body of comparative and randomised literature has challenged the assumption that non-weight-bearing is necessary after stable fixation, drawing on bone-biology evidence of the mechanical dependence of fracture healing.

At our institution, patients undergoing ORIF with stable plate fixation of Schatzker type V-VI fractures are permitted full weight-bearing from postoperative day 1. Reports of immediate weight-bearing specifically after fixation of Schatzker type VI fractures, the most unstable pattern, remain scarce, as most comparative studies pool this subtype within mixed cohorts. Here, we report three consecutive patients managed under this revised protocol, report their six-week outcomes, and discuss our findings against the literature on weight-bearing timing, with attention to the orthopaedic rationale for why early loading may help rather than harm.

## Case presentation

We report three consecutive patients treated at our institution for closed, high-velocity Schatzker type V-VI tibial plateau fractures. None had open wounds, compartment syndrome, or neurovascular compromise. All underwent preoperative radiographs and computed tomography (CT) with three-dimensional reconstruction. After an interval of 7-10 days, ORIF was performed using a lateral locking plate with a medial and/or posteromedial buttress plate, with allograft impaction of the metaphyseal void created after elevation of the depressed fragments where required. The final plate configuration was at the surgeon's discretion, depending on the metaphyseal apices and construct stability. Postoperative and six-week follow-up radiographs were obtained. In departure from the conventional non-weight-bearing protocol, all three patients were mobilised to full weight-bearing as tolerated from the first postoperative day, using a walking aid for balance rather than for offloading, with early active range-of-motion exercises and no brace. Functional outcome, comprising range of motion, pain during activities of daily living, and gait, was assessed clinically and by video of assisted walking at six weeks.

Case 1

A 39-year-old woman presented after a high-velocity road traffic accident with a closed, swollen left knee and inability to bear weight; she was neurovascularly intact with no associated injuries. Radiographs and CT confirmed a bicondylar Schatzker type V fracture with lateral condylar depression and comminution (Figure 1A-1D). She underwent ORIF with a three-plate construct comprising a lateral locking plate, a posterior plate, and a supplementary medial buttress plate, with allograft impaction of the void created after elevation of the depressed fragments (Figure 1E-1F). She was mobilised to full weight-bearing with a walking aid for balance from postoperative day 1. Continuous passive motion as tolerated was commenced twice daily from postoperative day 1, and active knee range-of-motion exercises were encouraged. At the six-week follow-up, the radiograph (Figure 1G-1H) showed maintained articular reduction with no implant failure or re-depression; she had regained knee flexion beyond 90 degrees, reported no pain during activities of daily living, and walked with a normal gait on video assessment.

**Figure 1:**
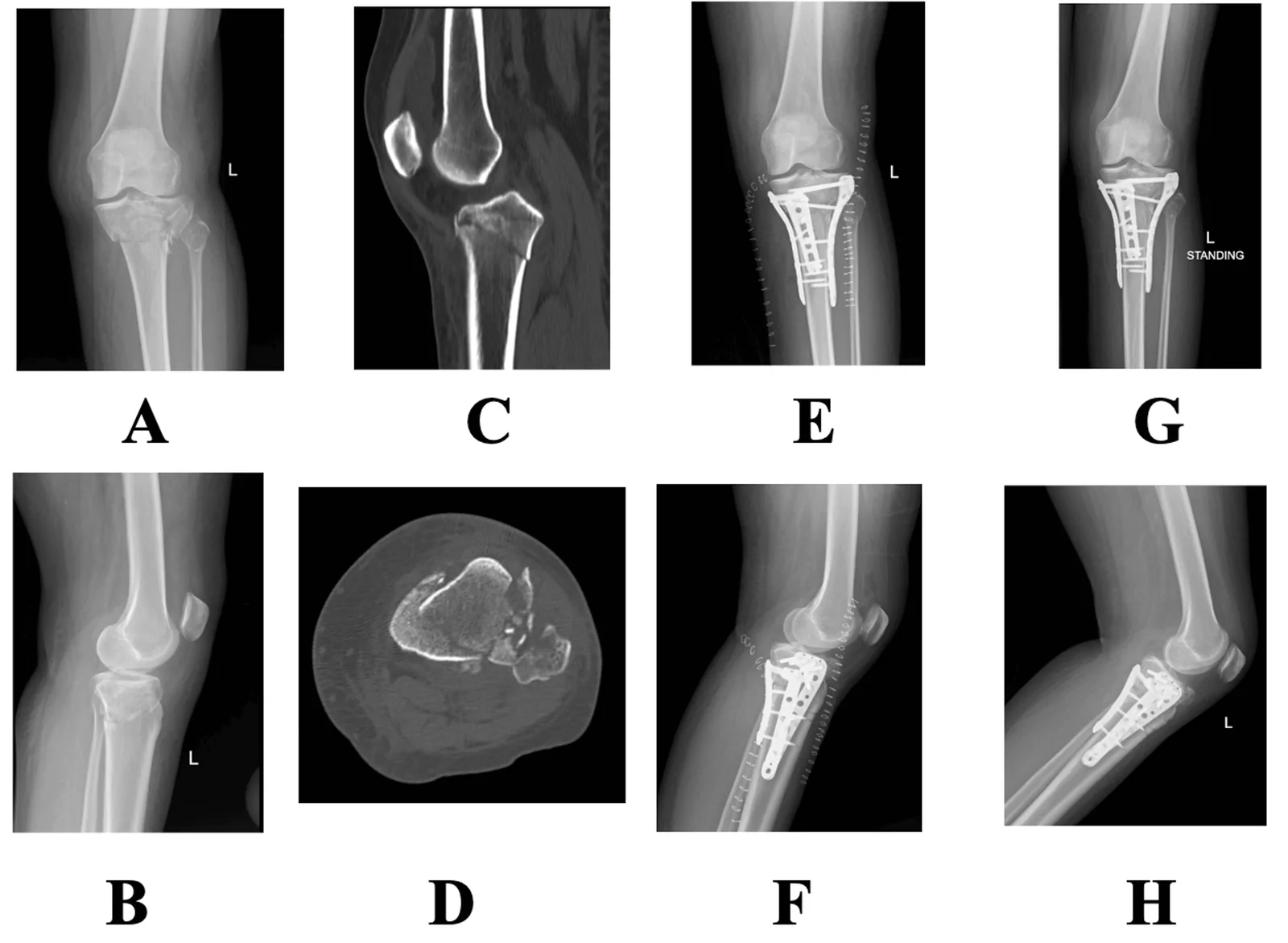
Case 1, 39-year-old woman, left bicondylar Schatzker type V fracture (A) Anteroposterior and (B) lateral radiographs showing a bicondylar Schatzker type V fracture with lateral condylar depression and comminution. (C) Sagittal and (D) coronal preoperative three-dimensional computed tomography reconstruction of the left proximal tibia and immediate postoperative (E) anteroposterior and (F) lateral radiographs showing three-plate fixation with a lateral locking plate, a medial buttress plate, and a posterior buttress plate. Standing (G) anteroposterior and (H) lateral radiographs at six weeks, obtained after full weight-bearing mobilisation from the first postoperative day, showing maintained articular reduction and early union with no implant failure or re-depression.

Case 2

A 72-year-old man was brought in after a high-velocity road traffic accident with a closed, deformed right knee; distal pulses and sensation were intact. Imaging confirmed a bicondylar Schatzker type VI fracture (Figure 2A-2D), the case of greatest a priori concern in this series regarding bone quality and tolerance of immediate loading. He underwent dual-plate fixation with a lateral locking plate and a posteromedial buttress plate, with allograft impaction of the void created after elevation of the depressed fragments (Figure 2E-2F), and was mobilised to full weight-bearing from postoperative day 1 under the same protocol. A standing radiograph at six weeks (Figure 2G-2H) confirmed maintained alignment without loss of fixation. He achieved knee flexion greater than 90 degrees, reported no pain during activities of daily living, and demonstrated a normal gait pattern on video assessment.

**Figure 2:**
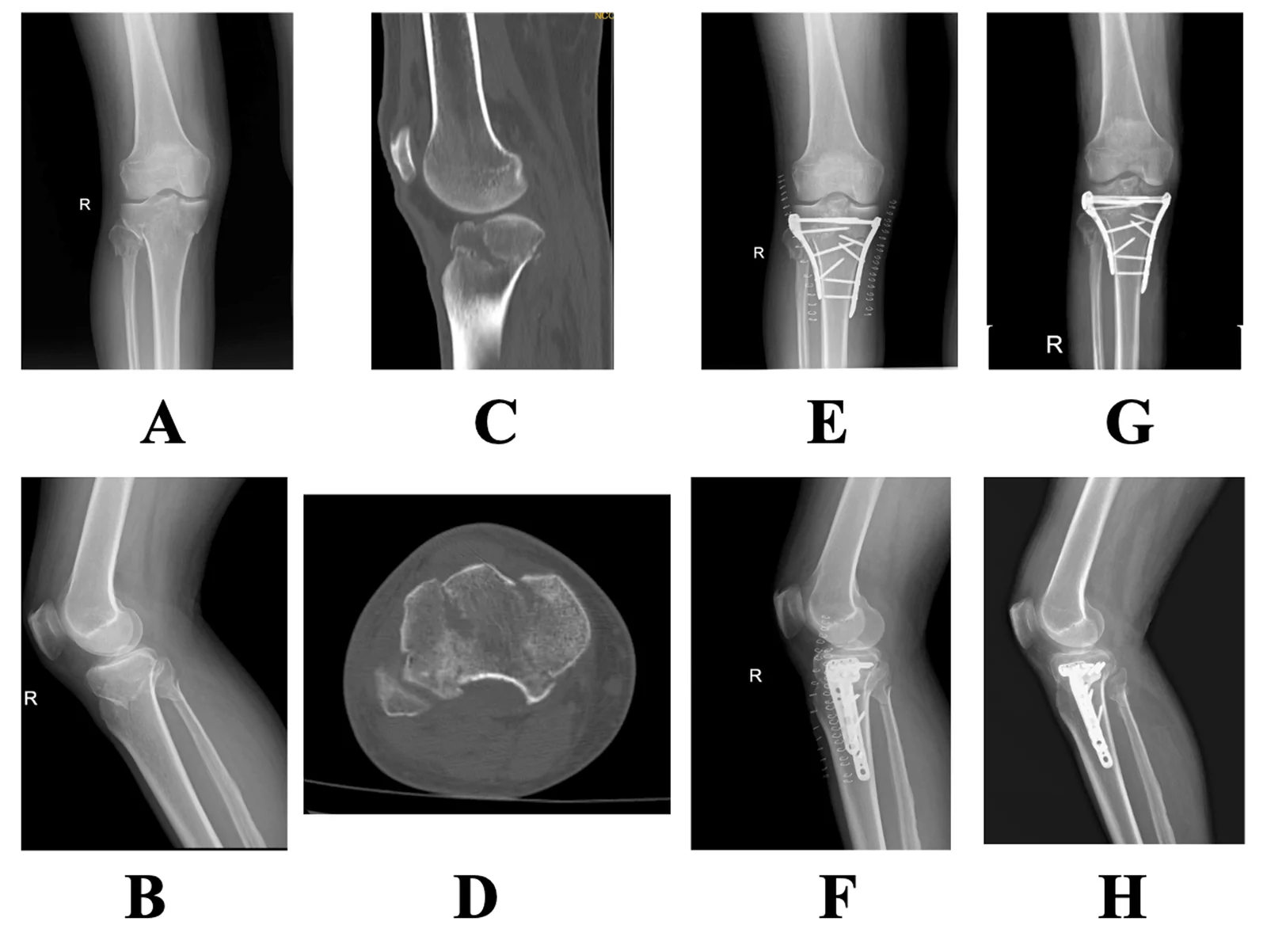
Case 2, 72-year-old man, right bicondylar Schatzker type VI fracture (A) Anteroposterior and (B) lateral radiographs showing a bicondylar Schatzker type VI fracture. (C) Sagittal and (D) coronal preoperative three-dimensional computed tomography reconstruction of the right proximal tibia. Immediate postoperative (E) anteroposterior and (F) lateral radiographs showing dual-plate fixation with a lateral locking plate and a posteromedial buttress plate. Standing (G) anteroposterior and (H) lateral radiographs at six weeks showing maintained alignment without loss of fixation, despite immediate full weight-bearing mobilisation.

Case 3

A 51-year-old man presented after a high-velocity road traffic accident with a closed right knee injury and was neurovascularly intact. CT confirmed a bicondylar Schatzker type VI fracture (Figure 3A-3D), managed with three-plate fixation and allograft impaction of the void created after elevation of the depressed fragments (Figure 3E-3F) and the same immediate full weight-bearing protocol from postoperative day 1. A standing radiograph at six weeks (Figure 3G-3H) confirmed maintained alignment without loss of fixation. On gait video assessment, he had regained knee flexion exceeding 90 degrees, reported no discomfort with activities of daily living, and walked with a normal gait, consistent with Cases 1 and 2.

**Figure 3:**
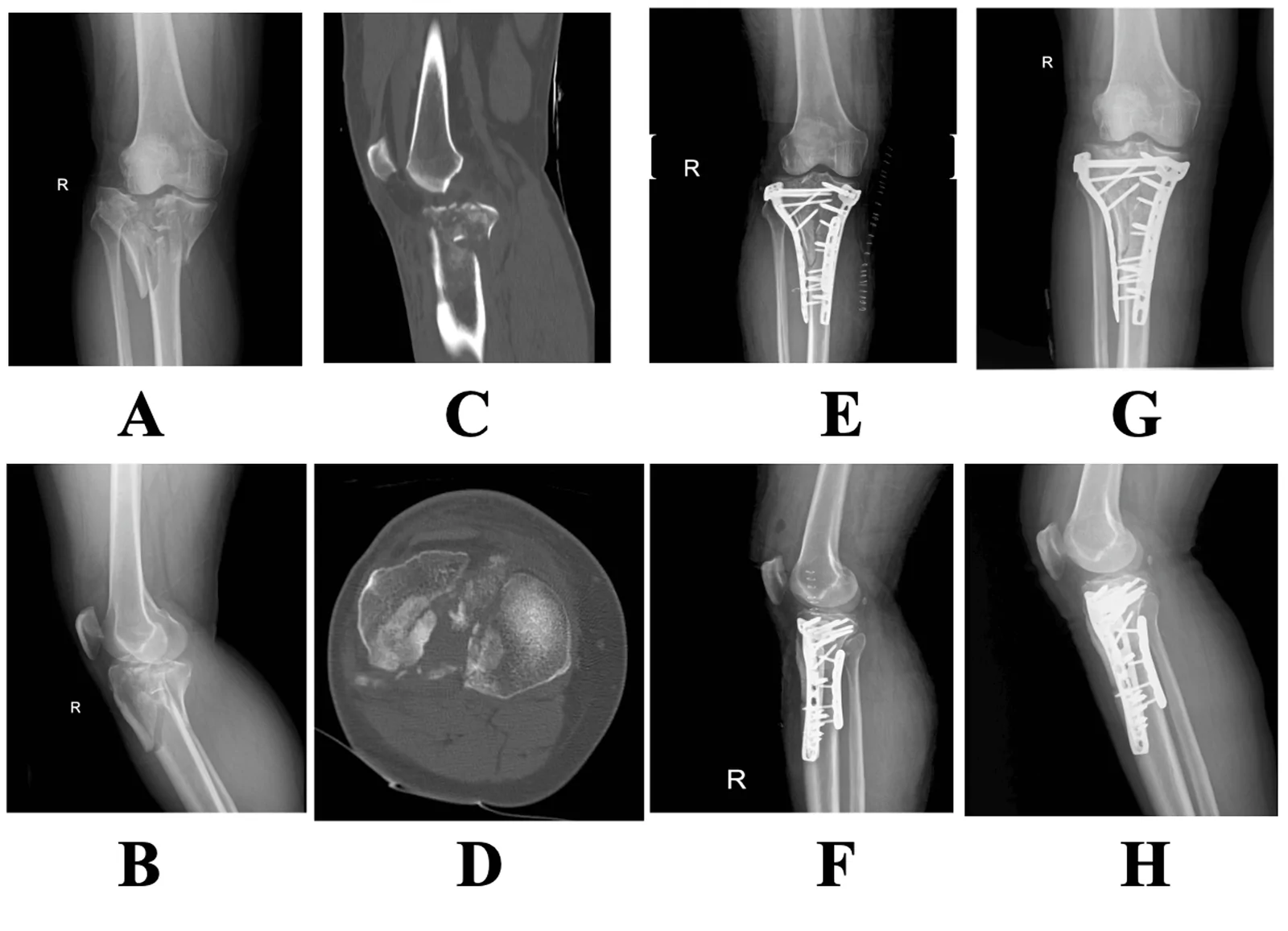
Case 3, 51-year-old man, right bicondylar Schatzker type VI fracture (A) Anteroposterior and (B) lateral radiographs showing a bicondylar Schatzker type VI fracture.(C) Sagittal and (D) coronal preoperative three-dimensional computed tomography reconstruction of the right proximal tibia. Immediate postoperative (E) anteroposterior and (F) lateral radiographs showing three-plate fixation with a lateral locking plate, a medial buttress plate, and a posteromedial buttress plate. Standing (G) anteroposterior and (H) lateral radiographs at six weeks showing maintained alignment without loss of fixation, despite immediate full weight-bearing mobilisation.

Across all three cases, no patient showed radiological loss of fixation or progressive articular depression, and none had a wound complication, infection, or revision surgery (Table 1).

**Table 1:** Summary of patient demographics, fracture and fixation characteristics, and six-week outcomes VAS: Visual Analog Scale; F: female; M: male; POD1: postoperative day 1; RTA: road traffic accident; ROM: range of motion; WB: weight-bearing

Case	Age/sex	Side	Mechanism	Fixation construct	Weight-bearing protocol	ROM (6 weeks)	VAS score (6 weeks)	Gait (6 weeks)	Loss of reduction
1	39/F	Left	High-velocity RTA	Lateral locking + medial buttress + posterior buttress	Full WB with walker support from POD1	0-95 degrees	2	Normal	None
2	72/M	Right	High-velocity RTA	Lateral locking + posteromedial buttress	Full WB with walker support from POD1	0-100 degrees	1	Normal	None
3	51/M	Right	High-velocity RTA	Lateral locking + medial buttress + posteromedial buttress	Full WB with walker support from POD1	0-95 degrees	2	Normal	None

## Discussion

This report describes three patients with bicondylar Schatzker type V-VI tibial plateau fractures mobilised to full weight-bearing from postoperative day 1 after ORIF, in departure from our unit's previous convention. All three achieved favourable outcomes at six weeks with no fixation failure or loss of reduction. The findings, while limited by small sample size, are directly comparable with a growing literature questioning the necessity of prolonged non-weight-bearing after tibial plateau fixation.

Comparison with the conventional non-weight-bearing paradigm

The traditional rationale for restricting weight-bearing rests on the assumption that axial load will cause loss of reduction before bony consolidation, underlying AO guidance of 6-8, and up to 12, weeks of toe-touch weight-bearing. The use of locking plates with secure angular stability has enhanced the mechanical construct's ability to resist displacement of the fracture reduction. However, the empirical basis for restriction is thin. Williamson et al., in the largest single-centre series to date (n=90, of whom 19 were Schatzker type VI), found no fixation failure and no significant difference in articular depression between groups at six weeks (p=0.36) [3]. Haak et al. similarly found no radiographic displacement with immediate weight-bearing (n=12) versus non-weight-bearing (n=20) [4]. A randomised trial by Ibrahim et al. (n=54) found that immediate weight-bearing produced significantly better Rasmussen scores, less pain, and superior range of motion at six months, with equivalent radiological outcomes [5]. A 2025 meta-analysis of 10 studies and 940 patients found no significant difference in Visual Analog Scale pain (standardised mean difference: 0.06; p=0.89), time to union (standardised mean difference: -0.45; p=0.32), or non-union (odds ratio: 0.14; p=0.10) between early and delayed weight-bearing [6], a finding echoed by an earlier systematic review of nine studies [7]. Our three Schatzker type V-VI cases, the pattern with the highest theoretical risk of construct failure under load, achieved outcomes fully concordant with this literature.

Not all evidence is reassuring. Stetson et al. described proximal tibial fractures after an abrupt, unplanned switch to full weight-bearing after tibial tubercle osteotomy [8], a reminder that immediate weight-bearing should be a deliberate, surgeon-directed decision based on intraoperative construct stability, not a default applied indiscriminately.

The orthopaedic and bone-biological rationale for early loading

Fracture healing is mechanically regulated. Wolff's law, refined into Frost's mechanostat concept, describes how bone remodels in proportion to strain: adequate loading stimulates osteoblastic formation, while unloaded bone resorbs [9]. Perren's strain theory describes how healing tissue stiffens as strain decreases, with bone formation proceeding only within a permissive strain range that is essentially absent in an unloaded construct [10]. Claes et al. showed that osteoblasts are stimulated dose-dependently by strains of 4-12% [11], and the unified bone-healing unit theory of Elliott et al. integrates these biological and mechanical factors in determining union [12], suggesting that non-weight-bearing may actively retard bone formation rather than merely fail to protect the fracture.

The cost of prolonged non-weight-bearing extends beyond the fracture site. Crutch-assisted non-weight-bearing increases the metabolic cost of ambulation up to fourfold [13], while prolonged immobilisation causes muscle-strength loss of 10-15% per week and increased thromboembolic risk [14]. Prescribed partial weight-bearing also shows poor compliance, with patients loading well beyond the instructed target [15], and Thewlis et al. found no association between weight-bearing ratio and one-year outcomes after tibial plateau ORIF [16]. These findings reframe immediate weight-bearing as a strategy with a positive physiological rationale, analogous to early mobilisation after hip fracture in the elderly.

In our series, allograft impaction of the metaphyseal void created after elevation of the depressed articular fragments provided additional structural support to the subchondral surface and, together with multi-planar plating, may have contributed to the stability that allowed immediate loading to be tolerated.

Patient selection and limitations

Immediate weight-bearing can reasonably be considered for tibial plateau fractures, including Schatzker type VI, provided the fixation is intraoperatively judged mechanically stable, typically with two or more plates for bicondylar patterns, and the decision remains individualised by comminution, bone quality, and reduction quality. Our cases should not be read as blanket support for unconditional immediate weight-bearing. This report is a small, retrospective report of three cases without a concurrent comparison group, with six-week follow-up only, and with functional outcome assessed clinically and by gait video rather than by a validated patient-reported outcome instrument. These are limitations to be addressed in future prospective work.

Clinical message

Prolonged non-weight-bearing after fixation of bicondylar tibial plateau fractures is a convention rather than an evidence-based necessity. Where the fixation construct is intraoperatively judged mechanically stable, unrestricted assisted full weight-bearing may be commenced on the first postoperative day, even in Schatzker type VI patterns and in elderly patients, without loss of reduction, and with the physiological benefits of early loading. The decision should remain a deliberate, surgeon-directed judgement of construct stability rather than a protocol applied indiscriminately.

## Conclusions

In these three patients with high-energy Schatzker type V-VI tibial plateau fractures treated with stable plate fixation, immediate full weight-bearing from the first postoperative day, in deliberate departure from conventional non-weight-bearing protocols, was associated with maintained radiological reduction, return to independent ambulation with a walking aid, knee flexion beyond 90 degrees, and freedom from pain at six weeks in every case, without any fixation failure or articular collapse. Although a report of three cases cannot establish causation, our experience supports a considered shift away from routine prolonged non-weight-bearing towards individualised, stability-guided early mobilisation, even for the most unstable Schatzker type VI pattern, provided the fixation construct is intraoperatively judged sound. These findings should now be confirmed in larger prospective comparative studies before immediate weight-bearing is adopted as routine practice.
